# Laryngeal embryonal rhabdomyosarcoma in an adult - A case presentation in the eyes of geneticists and clinicians

**DOI:** 10.1186/1471-2407-11-166

**Published:** 2011-05-12

**Authors:** Wojciech Kukwa, Piotr Wojtowicz, Beata Jagielska, Grzegorz Sobczyk, Andrzej Kukwa, Anna M Czarnecka

**Affiliations:** 1 Department of Otolaryngology, Czerniakowski Hospital, Medical University of Warsaw, ul. Stepinska 19/25, Warsaw, Poland; 2 The Maria Sklodowska-Curie Memorial Cancer Centre and Institute of Oncology, ul. Roentgena 5, Warsaw, Poland; 3 Laboratory of Molecular Oncology, Department of Oncology, Military Institute of Medicine, ul. Szaserow 128, Warsaw, Poland

## Abstract

**1. Abstract:**

**Background**

Rhabdomyosarcoma is a solid tumor, resulting from dysregulation of the skeletal myogenesis program. For rhabdomyosarcomas (RMS) with a predilection for the head and neck, genitourinary tract, extremities, trunk, retroperitoneum, the larynx is still an unusual site. Till now only several cases of this laryngeal tumor have been described in world literature in the adult population. The entire spectrum of genetic factors underlying RMS development and progression is unclear until today. Multiple signaling pathways seem to be involved in ERMS development and progression.

**Case presentation**

In this paper we report an interesting RMS case in which the disease was located within the glottic region. We report an embryonal rhabdomyosarcoma of the larynx in 33 year-old man. After unsuccessful chemotherapy hemilaryngectomy was performed. In follow up CT no signs of recurrence were found. Recently patient is recurrence free for 62 months.

**Conclusions**

Considering the histological diagnosis and the highly aggressive nature of the lesion for optimal diagnosis positron electron tomography (PET) and computerized tomography (CT) of the neck and thorax should be performed. At this time surgical treatment with adjuvant radiotherapy seems to be the treatment of choice for this disease. Rhabdomyosarcoma of the larynx has a better prognosis than elsewhere in the body, probably because of its earlier recognition and accessibility to radical surgery.

## 2. Background

More than 95% of laryngeal tumors in adults are squamous cell carcinoma. Laryngeal rhabdomyosarcomas are very rare, but extremely malignant tumors. Rhabdomyosarcoma must certainly rank close to, if not the least common of the laryngeal sarcomas. Most of reported cases have occurred in children [1]. Rhabdomyosarcoma arises from undifferentiated mesodermal tissue. It accounts for 40% of sarcomas found in the head and neck region. Among sarcomas arising in the larynx, rhabdomyosarcoma is one of the rarest. The first case of rhabdomyosarcoma of the larynx was reported by Glick in 1944 [2]. On gross pathology, rhabdomyosarcomas are soft, reddish-brown and lobulated. There are four principal histological varieties of rhabdomyosarcoma: embryonal, alveolar, pleomorphic and botryoid according to their degree of cellular differentiation and maturity. Adult rhabdomyosarcomas are usually composed of closely packed round cells with peripherally located nuclei. The cells have glycogen-rich, eosinophilic, vacuolated cytoplasm with cross-striations. Nevertheless they may have present cytological variability and resemble different stages of skeletal muscle morphogenesis. Immunostaining reveals positive findings for muscle-specific actin, desmin, and myoglobin and more specifically myogenin and MyoD1 are also positive [3]. Mitoses are typically absent [4]. Adult embryonal rhabdomyosarcoma has possibly unique molecular characteristics, nevertheless it has not been defined yet [5].

At the same time for oncology and surgery specialists the precise pre-therapeutical staging of tumors of the musculoskeletal system provides important prognostic information and has impact on the entire therapy management. During therapy imaging is extremely important in the follow-up and in diagnosing a possible recurrent disease [6]. Recently, PET and PET/CT have emerged as a functional diagnostic imaging modality for the management of various tumors in adult population and in the case of RMS FDG PET/CT is more accurate than CT regarding clinical staging and re-staging of patients with rhabdomyosarcomas. The overall TNM staging and M staging accuracies of FDG PET/CT was defined as high as 86% [7]. The specificity of PET/CT for the characterization of pulmonary metastases with a diameter > 0.5 cm and lymph node metastases with a diameter of <1 cm was significantly increased over that of CT alone [8].

Till now only several cases of this laryngeal tumor have been described in world literature in the adult population [9-11]. Although rhabdomyosarcoma of the larynx tends to be less aggressive than rhabdomyosarcoma elsewhere in the head and neck region [12], adult patients with laryngeal rhabdomyosarcoma often present at a later stage than other laryngeal tumors, mainly squamous cell carcinoma [9], which negatively influence the prognosis. One needs to remember that although laryngeal involvement by primary and metastatic rhabdomyosarcoma is very rare, however when occurs-it can result in life-threatening upper airway obstruction [13].

Rhabdomyosarcoma is a solid tumor, resulting from dysregulation of the skeletal myogenesis program. Four histological subtypes occur in RMS-embryonal (ERMS), alveolar (ARMS), pleomorphic and botryoid. The entire spectrum of genetic factors underlying RMS development and progression is unclear until today. While chromosomal rearrangements account for the majority of alveolar tumors, the genetic defects underlying the pathogenesis of embryonal type remain largely undetermined. Cytogenetically ERMS karyotype is disturbed, most frequently hyperdiploid, with extra copies of chromosomes 2, 7, 8, 11, 12, 13, and 20. Aneuploidies typical of sporadic E-RMS include gain of chromosomes 3, 8, 13 and loss of chromosomes 9, 14, X [14]. Although no consistent structural chromosomal alteration has been identified in ERMS [15], loss of heterozygosity at 11p15.5 is the most frequent genetic alteration in embryonal rhabdomyosarcoma [16]. Multiple changes affecting chromosome arms are being detected in ERMS cases, including gain or loss of specific regions harboring cancer progression-associated genes [17]. Aneuploidy and mitotic chromosomal instability observed in RMS was suggested to be a result of the overexpression of the AURKA (aurora kinase A) gene that is reported in all RMS tumors tested [18]. Other gene associated with RMS development is BUBR1 (budding uninhibited by benzimidazoles 1 homolog beta)-a key protein in the mitotic spindle checkpoint and potent inhibitor of Cdc20. Missense germline mutations of BUB1B were reported in patients with ERMS and supported the link between aneuploidy and cancer development [19].

Molecular genetics analyses have not only assisted in understanding the molecular mechanism in sarcoma pathogenesis but also demonstrated new relationships within different types of sarcomas leading to a more proper classification of sarcomas [20].

Multiple signaling pathways seem to be involved in ERMS development and progression. Array-based CGH analysis in primary RMS detected copy number changes of transcription factors such as MYC-related and the cytoskeleton and cell adhesion gene-laminin gamma-2 and also p21-activated kinase-1[18]. In the animal model interaction between HER family genes and the p53 pathway was shown to be involved in the origin of human rhabdomyosarcoma [21]. Moreover the pathogenesis of ERMS was also linked to deregulation of the hedgehog signaling pathway. Recent findings suggest that haploinsufficiency for the two tumor suppressor genes PTCH (patched (Drosophila) homolog (nevoid basal cell carcinoma syndrome)) and SUFU (suppressor of fused homolog)-which are both active in this signaling pathway-is frequent in ERMS development [22].

In the majority of ERMS cases screened, overexpression of MCL1 (induced myeloid leukemia cell differentiation protein) and MAP2K4 (mitogen-activated protein kinase 4) genes, both involved in cell viability regulation, was reported [17]. At the same time a few studies performed on series of embryonal tumors suggest that dysregulation of RAS function may be relevant to ERMS disease pathogenesis. NRAS (neuroblastoma RAS viral (v-ras) oncogene homolog) mutations are found in as high number as 20% of primary ERMS cases [23]. Another gene of RAS family - HRAS (GTPase HRas also known as transforming protein p21) - was reported to be found in the state of uniparental disomy (UPD) and/or mutated in multiple ERMS cases. It was even subsequently suggested that HRAS germline mutations are the first step in ERMS development and are followed by lost of the other HRAS allele and expression of mutant HRAS only [24].

Moreover FGFR1 (fibroblast growth factor receptor 1) overexpression was detected in all primary RMS tumors and cell lines tested. A hypomethylation of a CpG island upstream to FGFR1 exon 1 was identified, which suggests a molecular - epigenetic - mechanism of FGFR1 overexpression [25]. Recent studies have also shown a significant involvement of insulin-like growth factor (IGF) signaling components in the pathogenesis of rhabdomyosarcoma. High levels of insulin-like growth factor II mRNA (IGF-II) were detected in ERMS and increased transcriptional activity was confirmed to be result of AP-2 (activating protein 2) transcription factor overexpression. Moreover differential expression of IGF pathway genes can distinguish RMS subtypes, with IGF2 significantly more expressed in ERMS. In such cases it is possible that PAX3/FKHR fusion gene inhibits IGF2 expression [26]. Embryonal rhabdomyosarcoma is also characterized by high expression levels of PAX3 and PAX7. As PAX3 and PAX7 are known to play a role in the regulation of migratory events in embryogenesis, it is possible that the metastatic potential of ERMS is driven by PAX overexpression [27]. Moreover ERMS may overexpress CXCR4 and CXCR7 receptors that bind prometastatic alpha-chemokine stromal-derived factor-1 (SDF-1).

Not only proteins but also microRNAs (miRNA) have been implicated in RMS development and the clinical behavior of RMS. Muscle-specific miRNAs levels are lower in RMS compared with skeletal muscle but generally higher than in other normal tissues. Low miR-206 expression correlates with poor overall survival and is an independent predictor of shorter survival in metastatic embryonal RMS cases without PAX3/7-FOXO1 fusion genes. Low miR-206 expression also significantly correlates with high SIOP stage and the presence of metastases at diagnosis. High miR-206 expression strongly correlated with expression of genes involved in muscle differentiation and low expression is associated with genes linked to MAPkinase and NFKappaB pathway activation [28]. At the same time miR-183 is significantly overexpressed in RMS and is an important contributor to cell migration and has a potential oncogenic role through the regulation of 2 tumor suppressor genes, EGR1 and PTEN [29]. Moreover introduction of miR-1 and miR-133a into an embryonal rhabdomyosarcoma-derived cell line is cytostatic, thereby suggesting a tumor suppressor-like role for these myogenic miRNAs [30].

All data collected till now suggest the involvement of genes encoding cell adhesion, cytoskeletal signaling as well as transcriptional and cell cycle components in RMS tumorigenesis [18]. It is not possible to indicate specific steps in cell transformation and further research is needed to understand the molecular mechanism underlying RMS carcinogenesis, deregulation of proliferation and differentiation of skeletal myoblasts. We urge reporting of these rare tumors to enhance understanding the genetic component associated with this disease. Understanding of the molecular biology of sarcomas is leading towards development of newer and more effective treatment regimens [20].

## 3. Case presentation

33 years old male patient was referred to Department of Otolaryngology, Medical University of Warsaw, due to isolated longstanding hoarseness. At first visit weight was 98.5 kg, and height: 186 cm. The patient had no medical history (diagnoses or operations), was non-smoker and was not under any treatment.

**Figure 1:**
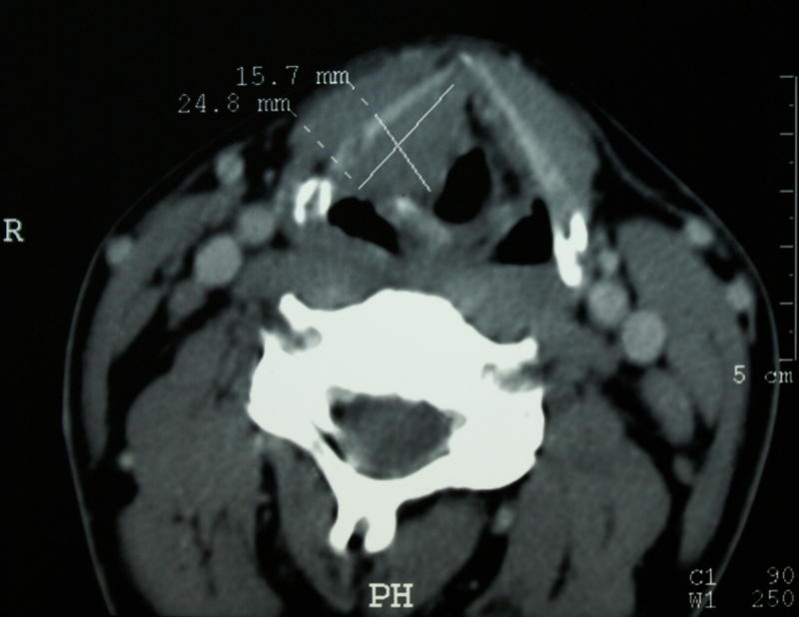
A two-phase CT scan, performed in August 2005 showing a 24.8 × 15.7 mm poorly delineated tumor, located at the right side of the larynx, invading the vocal fold and the ventricular fold, contracting the pyriform recess and constricting the glottis.

In May 2005, the patient underwent a direct laryngoscopy. During the procedure, samples of the tumor located in right side of glottis were collected for pathologic examination. Due to indistinct result of the first histo-pathological examination, the direct laryngoscopy was repeated in June 2005 at the Department of Otolaryngology of the Medical University of Warsaw, where the tumor was subsequently resected and examined by a pathologist. A second histopathological examination revealed fragments of edematous mucous membrane partially covered with columnar epithelium, nondysplastic and paraepidermal in majority. In the stroma, active granulation was identified. Moreover numerous "bizarre" pleomorphic stromal cells and proliferating epithelial cells were identified. Due to the lack of correlation between the result of the pathological examination and the clinical manifestation, the slides were sent to The Maria Sklodowska-Curie Memorial Cancer Centre and the Institute of Oncology in Warsaw for revision and additional specification. This consultation revealed *rhabdomyosarcoma embryonale*. For treatment planning in August 2005, computed tomography (CT) examination of the larynx was performed with contiguous 2.7 mm thick slices and showed a poorly delineated tumor located on the right side of the larynx, invading the vocal fold and the ventricular fold, contracting the pyriform recess and constricting the glottis. Contrast enhancement in the tumor was observed in the CT scans (Figure 1).

Due to the rarity of this type of tumor in laryngeal localization two cycles of VAC1 induction chemotherapy were administered (VAC1-vincristine, D-aktynomycine and cyclophosphamide: 1.5 MG/M2 IV day 1, 0.3 MG/M2 IV days 1-5, 150 MG/M2 IV days 1-5; every 28 days). The first chemotherapy cycle began on September 8^th ^2005. It was followed by chemo-radiotherapy in November and December 2005. Radiotherapy was administered in fractions of 200 cGy and total dose of 7000 cGy over 6 weeks. In parallel two cycles of maintenance VAC1 chemotherapy were given. CT was performed on the February 1^st ^2006 and revealed no tumor regression according to RECIST criteria. In accordance to CT scan no significant clinical response was observed. Subsequently on February 3^rd ^2006 vertical hemilaryngectomy was performed. Postoperative histopathological examination confirmed rhabdomyosarcoma located in the right side of the larynx. All examined margins were negative for cancer cells. The patient was discharged from the hospital with tracheostomy and nasogastric tube.

**Figure 2:**
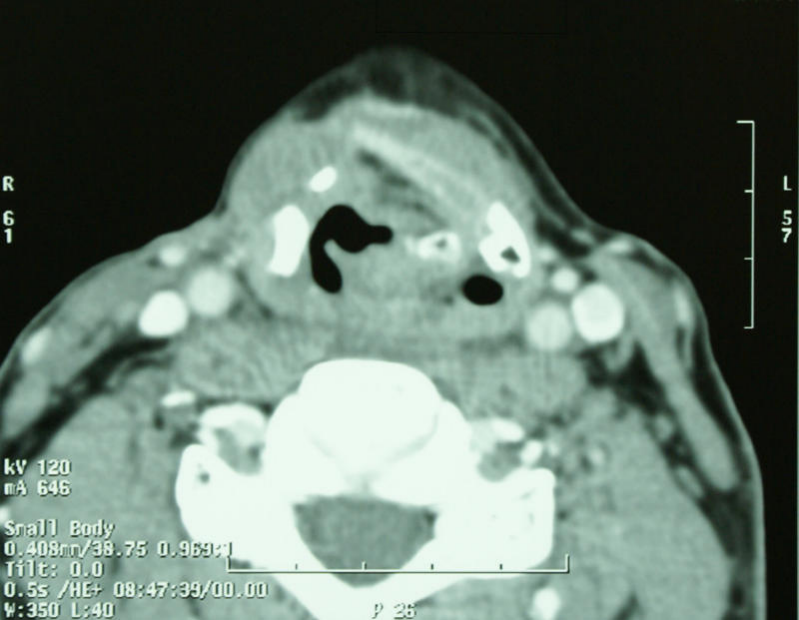
**A two-phase CT scan, performed in March 2009 showing the postoperative thyroid cartilage defect on the right side**. Soft tissue mass protruding into the supraglottic region on the left side in the aryepiglottic fold is identical to changes described in 2007 and 2008 (no signs of recurrence).

**Figure 3:**
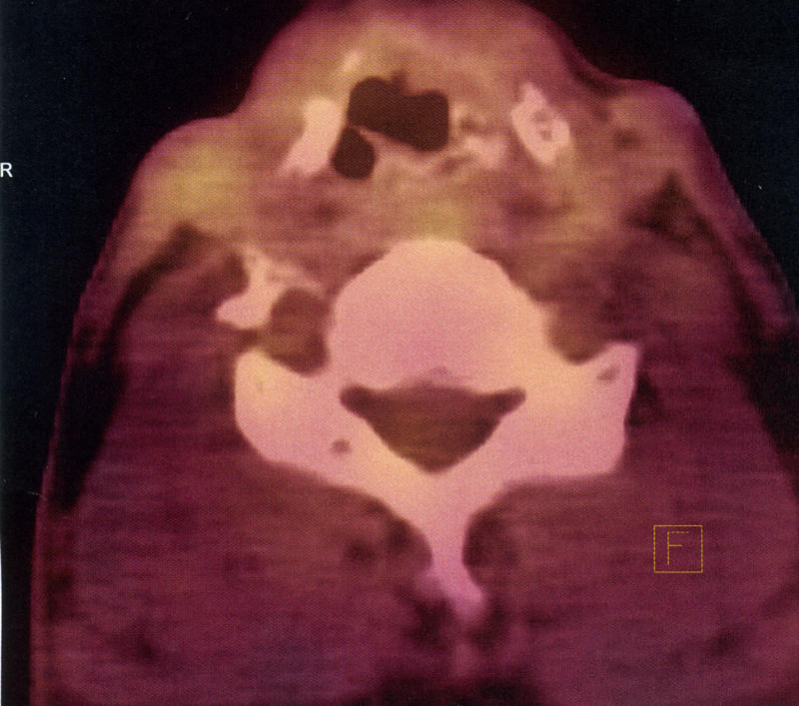
A PET/CT performed in June 2010 showing no local recurrence in the larynx.

First cycle of adjuvant chemotherapy was administered on April 21^st ^2006. Chemotherapy was continued until August 24^th ^2006. In total the patient received twelve cycles of VAC1, with 50% dose reduction after V cycle due to neutropenic fever in November 2005. The patient had the nasogastric tube removed in May 2006 and was decaniulated in October 2006. Following the surgery, the patient was monitored by a laryngologist and oncologist and underwent regular fiberoptic examinations of the larynx and direct laryngoscopy in case of unclear fiberoptic examination results. Follow-up CTs were performed in October 2006, June 2007, November 2007, June 2008 and March 2009, and did not reveal any tumor recurrence (Figure 2). Due to persistent swelling in the region of the arytenoid cartilage, a PET examination was performed on February 1^st ^2007. This examination did not reveal any focal lesions suggesting metastases. On March 2007, a check-up direct laryngoscopy with deep tissue biopsy was performed. The histopathological examination was negative. The patient remains under strict laryngological and oncological monitoring and no symptoms or signs of disease recurrence have been noticed. On June 24^th ^2010 patient had a follow-up PET-CT scan, which revealed normal glucose metabolism in the postoperative site (Figure 3). No metastases or local recurrence were found until now (May 2011).

## 4. Conclusions

Diagnosis of rhabdomyosarcoma may be difficult. Most of all adequate biopsy material is crucial in the identification of this tumor. The delay in diagnosis may arise as a consequence of diagnosis of inflammatory or benign laryngeal disease. The timely diagnosis of a laryngeal neoplasm depends on maintaining a high index of suspicion in a patient with progressive airway obstruction, dysphagia or dysphonia, and conducting an efficient work-up-including magnetic resonance imaging and direct laryngoscopy under general anesthesia in association with bronchoscopy-in order to define the extent of the lesion, rule out multiple lesions, establish and maintain an airway, and perform a biopsy of the tumor [31]. Emrbyonal rhabdomyosarcoma of the larynx must be differentiated from other common histologic types of larynx tumors in that it differs significantly with respect to its management. Rhabdomyosarcoma in adults is infrequent disease, and no treatment guidelines have been established. Only several of these tumors have been adequately described in world literature in the adult population. Adult patients with laryngeal rhabdomyosarcoma may present at a later stage than other laryngeal tumors, including squamous cell carcinoma. Treatment of rhabdomyosarcoma should be a multimodality effort. Although laryngeal RMS is an extremely rare tumor which responds to surgery, but also may be treated by means of chemo-radiotherapy. Further studies are needed in order to improve our understanding of its biological behavior and to define the most appropriate therapeutic approach. Considering the histological diagnosis and the highly aggressive nature of the lesion for optimal diagnosis positron electron tomography (PET) and computerized tomography (CT) of the neck and thorax should be performed.

At this time surgical treatment with adjuvant radiotherapy seems to be the treatment of choice for this disease. If possible initial efforts should be aimed at tumor size reduction, as unlike other regions of the body the head and neck region is limited by anatomic constraints and therefore, surgical treatment in this area is limited accordingly. Until recently, rhabdomyosarcomas carried a dismal prognosis; however, combined treatment with surgery, irradiation, and triple chemotherapy appears to have improved the outlook. This should probably be the treatment for laryngeal rhabdomyosarcomas. It would seem that a rhabdomyosarcoma of the larynx has a better prognosis than elsewhere in the body, probably because of its earlier recognition and accessibility to radical surgery.

## 5. Competing interests

The authors state no financial or non-financial competing interests (including political, personal, religious, ideological, academic, intellectual, commercial or any other).

## 6. Authors' contributions

AMC, WK-have been involved in drafting the manuscript, AK, WK, PW, BJ, GS-diagnosed and treated the patient, AMC, WK, AK-have made substantial contributions to the conception and design of the manuscript. All authors have read and approved the manuscript.

## Pre-publication history

The pre-publication history for this paper can be accessed here:

http://www.biomedcentral.com/1471-2407/11/166/prepub
